# Fatal Postpartum Hemorrhage in Diffuse Midline Glioma with H3-K27M Mutation

**DOI:** 10.1155/2019/8340437

**Published:** 2019-08-21

**Authors:** Takeshi Miyazaki, Masahiro Tsuji, Shinya Hagiwara, Toshiko Minamoto, Noriyoshi Ishikawa, Junko Hirato, Sumihito Nobusawa, Yasuhiko Akiyama

**Affiliations:** ^1^Department of Neurosurgery, Shimane University Faculty of Medicine, 89-1 Enya, Izumo, Shimane 693-8501, Japan; ^2^Department of Obstetrics and Gynecology, Shimane University Faculty of Medicine, 89-1 Enya, Izumo, Shimane 693-8501, Japan; ^3^Department of Pathology, Shimane University Faculty of Medicine, 89-1 Enya, Izumo, Shimane 693-8501, Japan; ^4^Department of Human Pathology, Gunma University Graduate School of Medicine, 3-39-22 Showa, Maebashi, Gunma 371-8511, Japan

## Abstract

Management of pregnant women with brain tumors necessitates difficult decision-making especially for estimating or preventing its intratumoral hemorrhage. A 26-year-old, 19-week pregnant woman complaining of headache and vomiting was admitted to our hospital. Magnetic resonance imaging (MRI) revealed hydrocephalus and a mass lesion without contrast enhancement extending from the left thalamus. To resolve severe symptoms, a ventriculoperitoneal shunt was inserted, and a biopsy was taken via the right ventricle. Pathological examination suggested diffuse or pilocytic astrocytoma, but subsequent genetic analysis revealed the diagnosis of midline glioma with H3-K27M mutation. The patient opted not to terminate the pregnancy, and MRIs conducted every four weeks revealed no change in tumor aspect. The patient delivered a healthy baby by cesarean section, and postpartum day 1 was uneventful. However, she was found in a coma due to a massive intratumoral hemorrhage on postpartum day 2 and died 3 weeks after the hemorrhage. This is the first case report of diffuse midline glioma with H3-K27M mutation in a pregnant woman followed by fatal hemorrhage. It highlights the necessity of careful clinical management and frequent neuroimaging during the entire perinatal period, even if the tumor has hypovascularity or low proliferative potential on radiological or pathological findings.

## 1. Introduction

There are few reports of pregnant woman with brain tumors associated with intratumoral hemorrhage. Therefore, clinical management or estimation of this condition has not been well-established. Furthermore, it is difficult to predict the potential for fatal intratumoral hemorrhage, especially in hypovascular and noncontrast material-enhanced tumors. Herein we report the first case of diffuse midline glioma with H3-K27M mutation in a pregnant woman followed by fatal hemorrhage during the postpartum period.

## 2. Case Presentation

**Figure 1:**
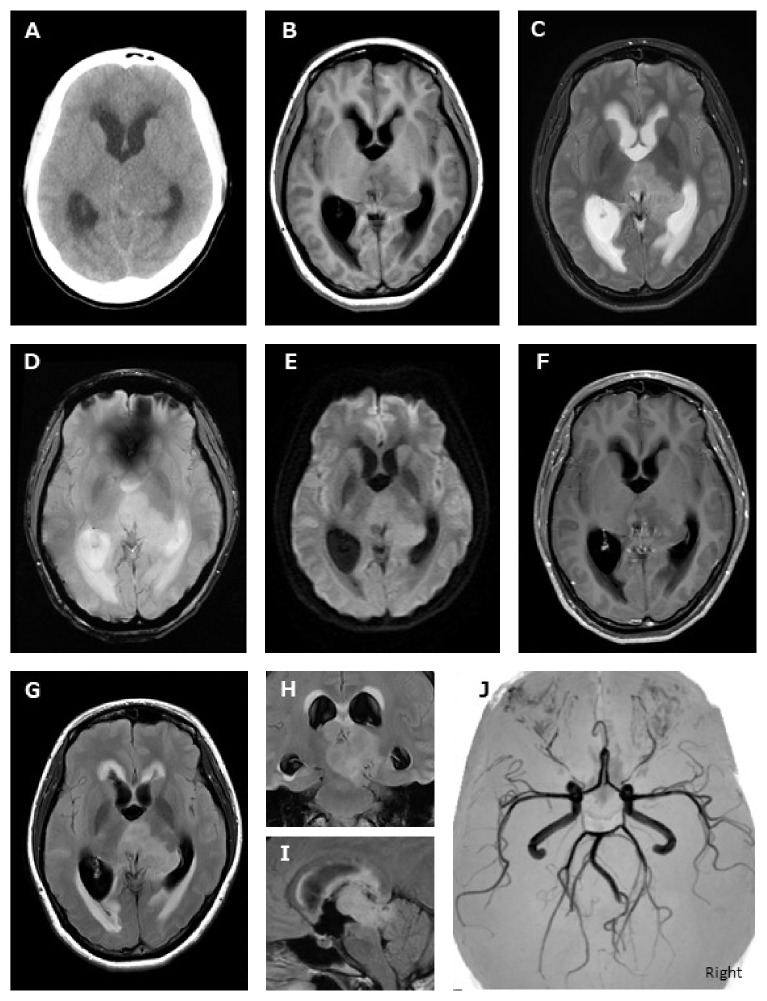
CT imaging revealed hydrocephalus and an iso-density mass lesion at the left thalamus (A). MRI revealed imaging revealed a poorly circumscribed mass lesion in the left thalamus, extending to both the midbrain and the right thalamus. The signal patterns were T1-low (B), T2-high (C), T2^*∗*^-high (D), diffusion weighted image-slight high (E), T1 Gadrinium-nonenhanced (F), and fluid attenuation inversion recovery-high (G; axial, H; coronal, I; sagittal), respectively. MRA showed no obvious vascular abnormalities (H).

A 26-year-old, and 19-week-pregnant woman consulted her obstetrician about intermittent headaches with vomiting. Hyperemesis gravidarum was suspected, and she was treated with antiemetic and analgesic drugs. However, symptoms gradually worsened, and she was admitted to the obstetric hospital. Computed tomography (CT) revealed hydrocephalus and an iso-density mass lesion in the left thalamus (Figure 1(A)), and the patient was transported to our hospital.

Magnetic resonance imaging (MRI) revealed a poorly circumscribed mass lesion extending to both the midbrain and the right thalamus from the left thalamus (Figures 1(B)–1(I)) and magnetic resonance angiography (MRA) detected no vascular abnormalities such as aneurysms, arterio-venous malformation or moya-moya disease (Figure 1(H)). The lesion was suspected to be low-grade glioma, because the intensity pattern of MRI imaging showed T1-low, T2-high, diffusion weighted image-slight high and T1 Gadrinium-nonenhanced morphology, respectively. After discussing the potential for a poor prognosis with caregivers, the patient and family opted not to terminate the pregnancy.

**Figure 2:**
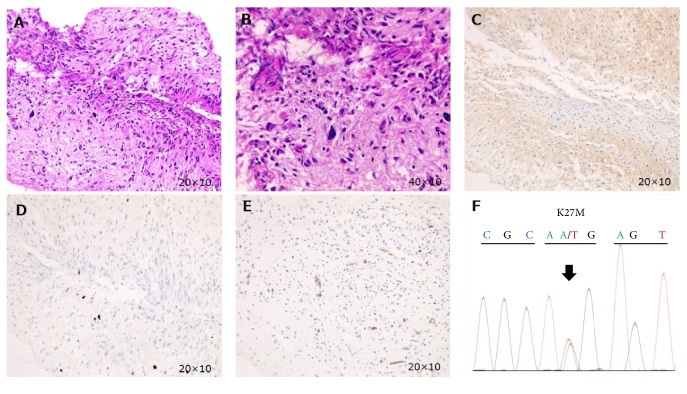
Hematoxylin-Eosin (H&E) staining revealed proliferation of various palisading cells with bipolar nature and no obvious findings of Rosenthal fiber (A, B). The immunohistochemical status of IDH1 showed wildtype IDH1 (C) and the Ki-67 labeling index was approximately 4% (D). CD34 staining suggested low microvascular proliferation (E). The optical magnification ratio of photomicrographs is 200-fold except for (B) (400-fold). The histone H3-K27M mutation was detected by Sanger sequencing (F).

The patient's symptoms continued to worsen, and she became unable to eat. To alleviate symptoms and confirm the diagnosis, a ventriculoperitoneal shunt was inserted, and a biopsy was performed via the right ventricle at the 21th week of pregnancy. The postoperative course was good, and symptoms resolved. Routine pathological examination of cell morphology suggested anaplastic astrocytoma, but the lack of necrosis or hypervascularity also suggested the possibility of diffuse astrocytoma or pilocytic astrocytoma with degeneration (Figures 2(A)-2(B)). Immunohistochemical isocitrate dehydrogenase 1 (IDH1) analysis suggested wildtype IDH1 status (Figure 2(C)). Moreover, immunohistochemical Ki-67 staining suggested low rate of cell proliferation (Figure 2(D)) and CD34 staining suggested low microvascular proliferation (Figure 2(E)). We therefore consulted another institution for definitive diagnosis and finally got the report that the H3-K27M mutation had been confirmed by Sanger sequencing (Figure 2(F)).

**Figure 3:**
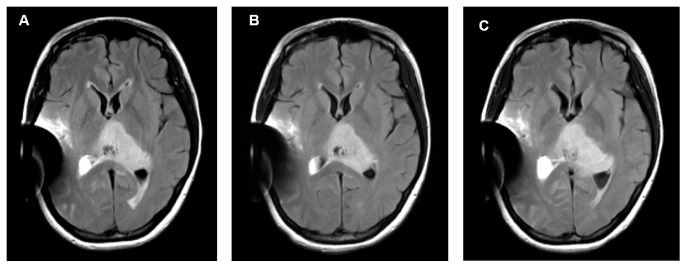
Follow-up MRIs conducted every 4 weeks revealed fluid attenuation inversion recovery and no change in tumor size and pattern of intensity ((A); shortly after surgery, (B); 4 weeks later, (C); 8 weeks later).

**Figure 4:**
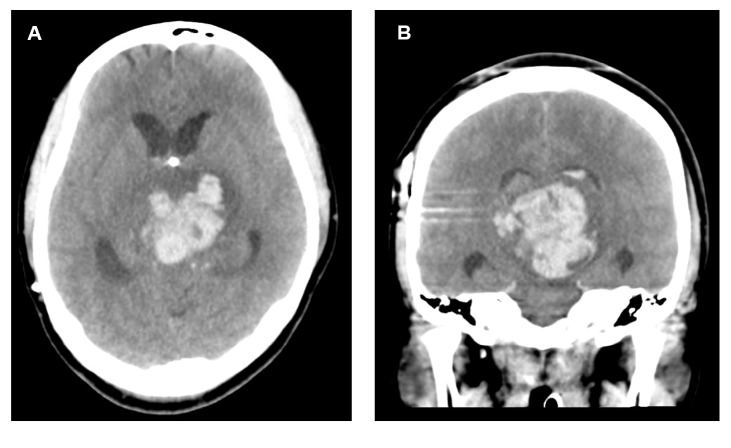
The emergency head CT on postpartum day 2 revealed cerebral herniation due to massive intratumoral hemorrhage ((A); axial view, (B); coronal view).

Follow-up MRIs conducted every 4 weeks showed no change in tumor size or intensity pattern (Figures 3(A)–3(C)). Clinical monitoring of the patient suggested a healthy pregnancy including no sign of pregnancy induced hypertension. A cesarean section was planned to facilitate a less-stressful and predictable delivery. The patient delivered a healthy baby by cesarean section with both spinal and epidural anesthesia at 38 weeks. The clinical course of postpartum day 1 was uneventful. The patient's systolic blood pressure was consistently near 100mmHg. However, she was found in a comatose condition on the floor next to her bed on the evening of postpartum day 2. An emergency head CT performed after resuscitation revealed cerebral herniation due to massive intratumoral hemorrhage (Figures 4(A)-4(B)), and the patient died 3 weeks after the hemorrhage.

## 3. Discussion

Diffuse midline glioma with H3-K27M mutation was newly established as a diagnostic category at the 2016 World Health Organization classification of the central nervous system and assigned to grade IV. However, it is difficult to diagnose diffuse midline glioma with H3-K27M through routine pathological examination because midline gliomas are widely diagnosed in various grades by many pathologists [1]. The diagnosis of diffuse midline glioma with H3-K27M mutation was time-consuming in our case, although a monoclonal antibody that detects the H3-K27M mutation recently became commercially available. Before receiving the genetic analysis, we suspected pilocytic astrocytoma with strong degeneration due to the low number of Ki67 positive cells, lack of necrosis or hypovascularity and nonenhanced MRI findings. We therefore chose observation until delivery. Had we been able to accurately diagnose the tumor earlier in the pregnancy, the patient would have had the option to terminate the pregnancy, or undergo chemotherapy while pregnant [2].

The incidence of tumor-related intracerebral hemorrhage has varied between studies, ranging from 0.6 to 5% [3–5]. Among primary brain tumors, gliomas are most prone to hemorrhage [4, 5]. Approximately 2.5% of astrocytomas are complicated by hemorrhage. Malignant astrocytomas are more likely to bleed [4, 6], although this can occur in astrocytomas of all histological grades [3]. Higher tumor grade or patient age of less than 14 is considered risk factors of intratumoral hemorrhage [7], but there is no report that midline glioma is more likely to be complicated by intratumoral hemorrhage. Only one case of adult brain stem low-grade glioma with intratumoral hemorrhage has been reported [8].

To our knowledge, there are few reports of pregnant woman with brain tumors followed by intratumoral hemorrhage. Prior reports include intratumoral hemorrhage of pituitary adenoma [9], optic glioma [10], choriocarcinoma [11], and chordoid meningioma [12]. These reports were unable to identify specific risk factors for intratumoral hemorrhage. In our case, there was no intratumoral hemosiderosis of T2^*∗*^ and no contrast material enhancement on gadolinium enhanced T1 on MRI. Further, immunohistological findings of CD34 staining indicated low tumor vascularity. We therefore assessed the risk of intratumoral hemorrhage as minimal, although we chose cesarean section as a precaution.

In the review of 75 cases of pregnant women with glioma, almost half of the cases chose vaginal delivery, and most were uneventful [2]. Therefore, the risk intratumoral hemorrhage may not be affected by the method of delivery. The relationship between capillary structure and hemorrhage in gliomas has been reported [6]. Recently, new MRI imaging techniques for detection of microvascular density, including susceptibility-weighted imaging and dynamic contrast-enhanced MRI, have become available [13, 14]. If these MRI images had been taken just before or after the delivery in this case, intratumoral microbleeds may have been detected. In that situation, we could have administered hemostatic agents and/or used general anesthesia followed by sedation for a few days to decrease the chance of postpartum intratumoral hemorrhage.

This is the first case report of diffuse midline glioma with H3-K27M mutation in a pregnant woman followed by fatal hemorrhage. Maximum clinical monitoring and frequent neuroimaging are necessary in pregnant women with brain tumors, especially at the perinatal period, even if the tumor has hypovascularity or low proliferative potential on radiological or pathological findings.
